# Inferring clocks when lacking rocks: the variable rates of molecular evolution in bacteria

**DOI:** 10.1186/1745-6150-4-35

**Published:** 2009-09-29

**Authors:** Chih-Horng Kuo, Howard Ochman

**Affiliations:** 1 Department of Ecology & Evolutionary Biology, University of Arizona, Tucson, Arizona 85721 USA

## Abstract

**Background:**

Because bacteria do not have a robust fossil record, attempts to infer the timing of events in their evolutionary history requires comparisons of molecular sequences. This use of molecular clocks is based on the assumptions that substitution rates for homologous genes or sites are fairly constant through time and across taxa. Violation of these conditions can lead to erroneous inferences and result in estimates that are off by orders of magnitude. In this study, we examine the consistency of substitution rates among a set of conserved genes in diverse bacterial lineages, and address the questions regarding the validity of molecular dating.

**Results:**

By examining the evolution of 16S rRNA gene in obligate endosymbionts, which can be calibrated by the fossil record of their hosts, we found that the rates are consistent within a clade but varied widely across different bacterial lineages. Genome-wide estimates of nonsynonymous and synonymous substitutions suggest that these two measures are highly variable in their rates across bacterial taxa. Genetic drift plays a fundamental role in determining the accumulation of substitutions in 16S rRNA genes and at nonsynonymous sites. Moreover, divergence estimates based on a set of universally conserved protein-coding genes also exhibit low correspondence to those based on 16S rRNA genes.

**Conclusion:**

Our results document a wide range of substitution rates across genes and bacterial taxa. This high level of variation cautions against the assumption of a universal molecular clock for inferring divergence times in bacteria. However, by applying relative-rate tests to homologous genes, it is possible to derive reliable local clocks that can be used to calibrate bacterial evolution.

**Reviewers:**

This article was reviewed by Adam Eyre-Walker, Simonetta Gribaldo and Tal Pupko (nominated by Dan Graur).

## Background

Dating the age of a lineage is fundamental to understanding evolutionary processes. This knowledge allows biologists to address questions concerning the ancestry and emergence of a lineage, its relationship to and coincidence with specific biological and geological events, the speed with which particular taxonomic groups have diversified, and the rates of molecular and phenotypic evolution [1,2]. In some cases, the age of a lineage can be determined directly from fossils [3,4]; but for the vast majority of life forms, the fossil record is either incomplete or entirely lacking, and insights into timing of evolutionary events must rely on other indirect methods.

This is largely the situation in bacteria. Despite being the most ancient, abundant and diverse group of organisms on the planet, bacteria have virtually no fossil record due both to their size and to the lack of fossilizable components. As a result, molecular clocks have been widely applied to estimate divergence times in bacteria [1,2,5,6], as well as in other organisms [7,8]. By assuming that nucleotide or amino acid substitutions accumulate at a fairly constant rate across taxa over evolutionary timescales, the association of one bacterial lineage to an event that occurred at a known time in the geologic record can yield the age of all other lineages by extrapolation. For example, aerobic bacteria can be linked to a time when atmospheric conditions was sufficient to support aerobic life; and similarly, obligate pathogens could not have evolved prior to the appearance of their hosts. Unfortunately, because there may be a gap between the availability of the requisite resource and its exploitation by bacteria, such events provide only an upper bound to the date of appearance of a bacterial lineage and are therefore of limited used for calibrating the molecular clocks. Furthermore, calibrating molecular clocks using events that have occurred in the distant past is complicated by the issue of large confidence intervals and rate heterogeneity [8].

A similar, but much more accurate, procedure for calibrating molecular clocks is based on the coincidence of speciation events between obligate symbionts and their eukaryotic hosts [9,10]. As observed in several primary endosymbionts of insects, there is complete concordance between the molecular phylogenies of the bacteria and their hosts [9,11-18]. This concordance is the results of strict vertical transmission and permits the unequivocal application of the hosts' fossil record to their endosymbionts, thereby providing the accuracy of fossil-derived dates to bacterial evolution. But because these endosymbionts have been shown to accumulate substitutions at a faster rate than do some free-living bacteria [10], certain corrections might be required to assign dates to particular groups of bacteria.

An alternative way to calibrate molecular clocks for estimating the age of bacterial lineages is to determine directly the rates at which mutations accumulate in experimental systems [19-21]. Despite the appeal of this method, such empirical estimates of mutation rates are of rather limited use when inferring the age of most bacterial lineages. First, the rates derived from mutation are usually calculated on a 'per generation' basis and are difficult to convert to actual time because the number of generations per year in natural populations or over evolutionary time scales is not known. Moreover, laboratory-derived mutation rates can differ from one another, and from those in natural habitats, by orders of magnitude, thus rendering the extrapolation to broad evolutionary time scales unreliable [22,23].

The purpose of this paper is to determine the extent to which bacterial divergence times can be derived from molecular data. Using a series of internal and external calibration points, we ask if there is any gene or set of sites that can serve as a reliable molecular clock, and whether the molecular characters themselves portray a consistent view of bacterial evolution. Finally, we discuss which applications of bacterial molecular clocks are valid and justified or whether such attempts might be better abandoned.

## Results

### Calibrating rates of 16S rRNA divergence

Due to its universal distribution and slow rate of sequence evolution, 16S rRNA has been the most widely used gene for the identification and taxonomic assignment of bacteria, with nearly one million sequences representing all known bacterial diversity [24]. Our first goal was to determine if the rate of 16S rRNA sequence evolution could serve as a reliable molecular chronometer. To test this, we focused on bacterial endosymbionts whose ages have been established from the fossil records of their hosts [9,11-13,16,25-27].

**Figure 1:**
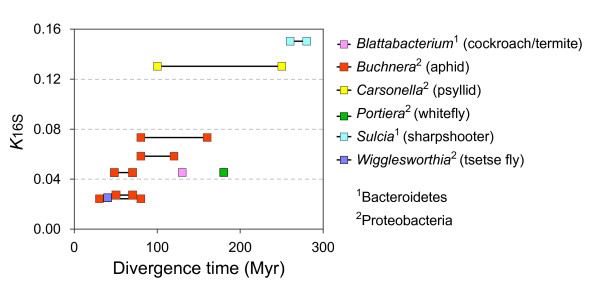
**Rates of 16S rRNA divergence in obligate endosymbionts**. Divergence times were estimated from the fossil record of insect hosts (see Methods). For *Buchnera*, *Carsonella*, and *Sulcia*, linked points denote the minimal and the maximal dates of divergence.

Figure 1 presents the extent of 16S rRNA sequence divergence (*K*_16S_) relative to the fossil-derived divergence times for six lineages of obligate bacterial endosymbionts infecting three orders of insects. Evolutionary rates vary approximately four-fold across all bacterial lineages considered (0.025 to 0.091% per million years) but appear to be fairly consistent for members within a clade. Estimates from the *Buchnera*-aphid association, for which the most abundant and accurate data are available, varied from 0.05 to 0.08% per million years, with an average of 0.06% per million years. Compared to their insect hosts, the rates of 16S rRNA divergence in endosymbiont lineages are highly variable. Applying the same fossil-derived divergence times, the 16S rRNA genes in *Buchnera *evolve 36 times faster than the analogous gene (*i.e*., 18S rRNA) in their insect hosts [10]; however, in the *Blattabacterium*-cockroach/termite association, the small subunit RNA genes of the endosymbionts and their hosts evolve at roughly the same rate [13].

### Sequence divergence in protein-coding genes

Based on their evolutionary distance, we selected 42 pairs of bacterial genomes (Additional file 1) to evaluate variation in substitution rates across genes and lineages. The genome pairs represent eight bacterial phyla and include strains or species that are sufficiently closely related to obtain unambiguous sequence alignments and reliable estimates of substitution rate. Remembering that up to a 3% difference in 16S rRNA genes is the usual cut-off for assigning bacteria to the same species [28], the *K*_16S _values between members of a genome-pair range from 0.07% to 3.4% (average = 0.98%).

As alternative measures of sequence divergence, we calculated the synonymous (*K*_s_) and nonsynonymous (*K*_a_) substitution rates for all protein-coding genes shared between the two members of a genome-pair; to avoid the distortion caused by genes that are under strong positive or purifying selection, we used the median value as the genome-wide level of sequence divergence. As expected from genome-pairs spanning such a broad range of 16S rRNA divergences, median *K*_s _values ranged from 0.11 to 1.29 (average = 0.6), and median *K*_a _values, from 0.006 to 0.058 (average = 0.03). In those genome pairs whose members were most divergent, there was much greater variation in *K*_a _and *K*_s _values across genes, which is due both to stochastic factors and to the metrics of multiple hit corrections.

**Figure 2:**
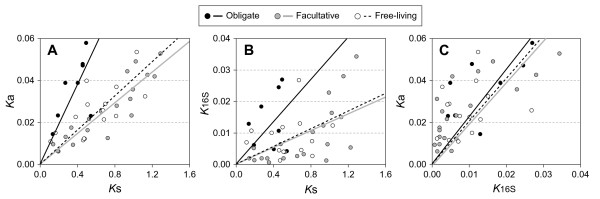
**Relationships among three measures of sequence divergence**. Bacteria constituting each genome-pair were classified according to their type of association with eukaryotic hosts. (A) Relationship between genome-wide nonsynonymous (*K*_a_) and synonymous (*K*_s_) site divergence. (B) Relationship between 16S rRNA divergence (*K*_16S_) and genome-wide *K*_s_. (C) Relationship between genome-wide *K*_a _and *K*_16S_.

To determine if these commonly used measures of bacterial sequence divergence - *K*_16S_, *K*_s_, and *K*_a _- increase in a correlated manner across all organisms (and thus provide largely consistent measurements of divergence times), we plotted their values against one another for the 42 genome-pairs. Because the evolution of 16S rRNA and at nonsynonymous sites of protein-coding regions are under selective constraints, both *K*_16S _and *K*_a _will be affected by the level of genetic drift, which underlies the accumulation of slightly deleterious mutations. For this reason, we grouped lineages according to particular lifestyles that are known to give rise to different levels of genetic drift (Figure 2).

As noted previously [*e.g*., [29,30]], bacteria that form obligate associations with eukaryotic hosts have significantly higher *K*_a_/*K*_s _ratios (Figure 2A, ANOVA test P-value = 8.0 × 10^-9^), a consequence of their smaller effective population sizes (*N*_e_) compared to bacteria that are free-living or form facultative associations. Likewise, rates of 16S rRNA evolution are elevated in obligate pathogens and symbionts (Figure 2B, ANOVA test P-value = 7.2 × 10^-4^), as resulting from increased levels of genetic drift. Because the effects of genetic drift are genome-wide, both *K*_a _and *K*_16S _are similarly affected by reductions in *N*_e_, and therefore, *K*_a_/*K*_16S _ratios are not expected to differ significantly between the three lifestyle-groups (Figure 2C, ANOVA test P-value = 0.37).

As presented in Figure 2C, nonsynonymous sites evolve, on average, at about twice the rate of 16S rRNA, but the broad scatter of points indicates that this is not a general trend across genomes. This lack of a consistent association implies that divergence times obtained by applying a molecular clock to one of these measures of sequence divergence will rarely match those calibrated to another. There is, however, a fairly reliable relationship between *K*_a _and *K*_s _within each of the lifestyle groups. Looking across the lineages considered, synonymous sites evolve 25 times faster than do nonsynonymous sites in free-living bacteria, but only 10 times faster in bacteria that form obligate associations with eukaryotic hosts.

### Core Gene Evolution

**Figure 3:**
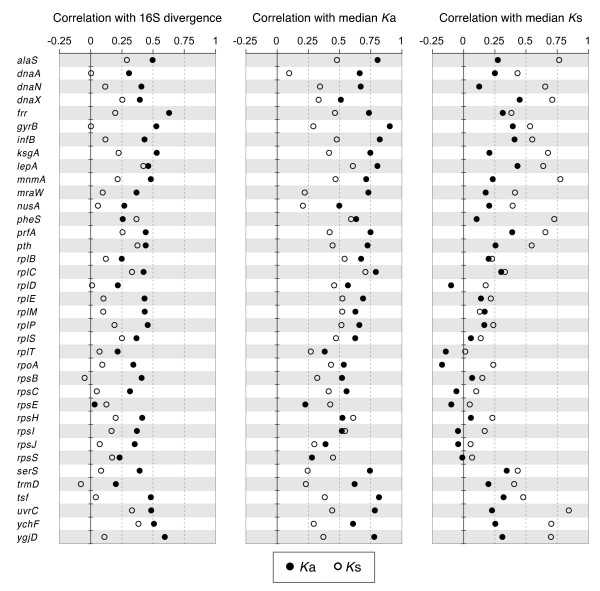
**Patterns of sequence evolution in universally conserved genes**. Concordance between synonymous and nonsynonymous site divergence in 37 genes and other measures of sequence divergence. Left panel: *K*_a _and *K*_s _*vs*. 16S rRNA divergence. Center panel: *K*_a _and *K*_s _*vs*. genome-wide median nonsynonymous divergence. Right panel: *K*_a _and *K*_s _*vs*. genome-wide median synonymous divergence.

Aside from 16S rRNA, several universally conserved genes have been used to determine the relationships among bacteria [1,2,31,32]. Although neither the genome-wide median *K*_a _nor *K*_s _values show a strong association with 16S rRNA divergence (Figure 2), it is possible that particular genes evolve in a fashion that would make suitable molecular clock. We examined 37 single-copy genes present in all of the 82 genomes sampled (Additional file 2), estimated the *K*_s _and *K*_a _for each gene, and determined the correlation of each with median *K*_s _and *K*_a _values and with 16S rRNA divergence. As observed for the genome-wide estimates, there is usually a much better correlation in between *K*_a _and *K*_16S _than between *K*_s _and *K*_16S _(Figure 3). In fact, for 90% of the universally conserved single-copy genes, there is no significant relationship between their divergence at synonymous sites and divergence of 16S rRNA. The situation differs from that observed between divergence at individual genes and the median *K*_s _and *K*_a _values; as expected, individual genes are generally significantly correlated with their respective median values. However, there is a notable exception: In every ribosomal protein (Rpl and Rps) considered, neither the *K*_a _*nor the K*_*s *_values is significantly correlated with median *K*_s_. Although the genes encoding ribosomal proteins are short and might be subject to stochastic variation, the more likely reason that these genes depart from the majority of genes in a genome is because their sequences are highly conserved at both nonsynonymous and synonymous sites due to constraints on protein function and to codon usage bias, respectively.

## Discussion

Bacteria have played a vital role in shaping life on Earth throughout their long history of evolution. Although accurate timescales have been fundamental to studying evolutionary processes, there is, with the exception of very few milestones, virtually no fossil record by which we can chronicle the history and diversification of bacteria. This circumstance has resulted in the broad application of molecular clocks to obtain divergence times, and in this paper, we investigated the extent to which molecules are accurate chronometers of bacterial evolution.

The short answer is that no gene, nor class of sites within genes, can serve as a reliable molecular clock for bacteria. Despite early claims of similar substitution rates among bacteria [5,9] and the application of eukaryotic substitution rates to bacteria [*e.g*., [33]], no single evolutionary rate can be applied across diverse bacterial lineages or over broad evolutionary periods. We first applied the most direct approach by evaluating rate constancy for the 16S rRNA genes of insect endosymbionts whose divergence times are echoed in the fossil record of their insect hosts. Although the 16S rRNA gene homologs of aphid endosymbionts were originally estimated to evolve at a rate only slightly faster than that obtained for free-living bacteria (*i.e*., 1-2% per 50 million years), the inclusion of several additional taxa now provides clear evidence of much wider variation. When considering only insect endosymbionts, whose intercellular lifestyles might be thought to engender similar selective constraints and similarly fast mutation rates, we detected nearly a 4-fold difference in 16S rRNA gene substitution rates. This difference in rates is not due to vagaries in the fossil record: in the case of sharpshooter insects, which contain obligate co-symbionts within the same host, the 16S rRNA genes of the gammaproteobacterial *Baumannia *evolve at nearly five times the rate of homologs in the bacteroidales *Sulcia *[17].

If 16S rRNA genes cannot serve as a reliable chronometer for bacterial evolution, perhaps there are other genes that evolve in a more consistent manner. The original molecular clock hypothesis [34] was formulated based on the accumulation of amino acid variants in proteins. On a genome-wide scale, nonsynonymous substitution rates are strongly correlated with those in 16S rRNA genes, indicating that use of this class of sites is as problematic as 16S rRNA genes. Note that the relationship between *K*_a _and *K*_16S _is the same for bacteria of different lifestyles (statistically indistinguishable slopes of lines in Figure 2C), so despite the lineage-specific differences in mutation rates, population sizes, generation times and genetic drift, the population-level parameters affecting each group exert comparable effects on the evolution of 16S rRNA and nonsynonymous sites.

In theory, the synonymous sites of protein-coding genes reflect the underlying rate and pattern of mutation, and are not affected by either selection or genetic drift. This is apparent in the plots of *K*_s _against both *K*_a _(Figure 2A) and *K*_16S _(Figure 2B), in which those bacteria subject to higher levels of drift have elevated divergence at slightly deleterious sites (*K*_a _and *K*_16S_) relative to neutral sites (*K*_s_). However, mutation rates are not the same in all bacteria [22], and are known to vary within lineages depending on growth conditions [35] and within genomes depending on the chromosomal location or transcriptional status of a gene [36-38]. Moreover, synonymous sites reach saturation relatively rapidly [39,40] and, in many genes, are under selective constraints for translational efficiency [41] or mRNA secondary structure [42,43], all of which limit their utility as molecular clocks.

There appears to be no panacea when attempting to assemble a timescale for bacterial evolution. The high level of variation in substitution rates across genes and lineages suggests that no single molecule can ever serve as a universal clock in bacteria. Furthermore, the lack of clear calibration points, particularly for free-living bacteria, remains a major challenge in dating bacterial evolution. However, there is some hope: the example of the *Buchnera*-aphid association has demonstrated that estimating divergence times based on a local, more lineage-specific clock is feasible [9]. Because the consistency of substitution rates in gene or protein can be tested by a relative-rate test [44,45], it is possible to determine whether particular genes evolve at a uniform rate for a set of lineages (but see [46] for the limitations). Once a reliable phylogeny is produced, the use of a single calibration point will provide, by extrapolation, estimates of divergence time for all other lineages. So while we oppose the indiscriminate application of a single rate to calibrate the whole of bacterial history, it is possible to make robust statements about bacterial divergence times and to calibrate key dates in bacterial evolution.

## Methods

### Data source and genome-pair selection

To examine substitution rates across loci and taxa, we selected 42 pairs of bacterial genomes that encompassed a range of genome-wide average Ks values from 0.19 to 1.02 [29]. Genome sequences were downloaded from NCBI GenBank [47] on October 1, 2008; genome project ID and species names of the 84 genomes are listed in Additional file 1. Data parsing and processing were performed with a set of custom Perl scripts written with Bioperl modules [48].

### Sequence divergence of 16S rRNA genes

To calibrate rates of 16S rRNA divergence (*K*_16S_) in bacteria, we obtained the estimates of sequence divergence and fossil-based divergence times from the literature [9,11-13,16,25-27]. For each of the 42 selected genome-pairs, we calculated the *K*_16S _between their constituent members by first aligning the nucleotide sequences in MUSCLE [49] using the default parameters and then applying the DNADIST program in the PHYLIP package [50] to calculate the level of sequence identity. In cases where there are multiple 16S rRNA genes within a single genome, we performed all possible pair-wise comparisons of 16S rRNA genes between both members of the genome-pair and used the median value to denote the level of divergence.

### Orthologous protein-coding gene identification

To identify orthologous protein-coding genes between the members of a genome-pair, we performed reciprocal BLASTP [51] sequence similarity searches on every annotated protein sequence in the two genomes. A pair of genes were defined as orthologs in the two genomes if: (1) the protein sequences were reciprocal best-hits, (2) the BLASTP E-value was less than or equal to 1 × 10^-15^, (3) the difference in length was no more than 20% of the shorter sequence, (4) the high-scoring pair (HSP) accounted for at least 80% of the shorter gene, and (5) the amino acid sequence similarity was at least 90% within the HSP. The close relationship of the genomes within each of the 42 selected pairs coupled with the high stringency of our ortholog selection minimized, if not entirely eliminated, the inclusion of paralogs in the subsequent substitution rate calculations.

### Defining the core gene set

To define a set of conserved genes for cross-taxa comparison, we used OrthoMCL [52] to identify orthologous gene clusters among the 42 genome-pairs. Only single-copy genes that were shared by all 84 genomes were considered. The set of 37 genes recovered from this procedure are listed in Additional file 2.

### Substitution rate calculations

To calculate the synonymous (*K*_s_) and nonsynonymous (*K*_a_) substitution rates between pairs of orthologous protein-coding genes, we aligned the amino acid sequences in MUSCLE [49] using default settings. The resulting protein alignments were reverse-translated to codon-based nucleotide alignments with PAL2NAL [53]. Because many highly reduced genomes have a strong base compositional bias, we applied the YN00 method [54] implemented in the PAML package [55] to estimate the substitution rates. The mutation model used in the YN00 method accounts for the biases in base composition, codon usage, and transition/transversion rate.

### Statistical analyses

To test if the lifestyle of a bacterial lineage is a significant factor in determining the correlation between divergence rates, we separated the 42 sampled genome-pairs into three groups: free-living, facultatively host-associated, and obligately host-associated. The type of lifestyle was used as the independent variable in the analysis of variance (ANOVA) model implemented in the R statistical package.

## Competing interests

The authors declare that they have no competing interests.

## Authors' contributions

HO and CHK conceived of the project; CHK carried out all of the analyses; HO and CHK interpreted the data and drafted the manuscript; HO nitpicked about stylistic issues. Both authors read and approve of the submitted manuscript.

## Reviewers' comments

### Reviewer's report 1

Dr Adam Eyre-Walker, University of Sussex, Brighton, United Kingdom

#### Reviewer comments

In this interesting manuscript Kuo and Ochman investigate whether the rate of molecular evolution in bacteria is constant across lineages. Unfortunately, measuring the rate of evolution is generally difficult in bacteria because they have no fossil record. This leaves two alternatives, relative rate tests and using the fossil record of the host of vertically transmitted endosymbionts. The authors employ this latter strategy. They find that rates of 16S rRNA evolution vary by about 4-fold across different groups of bacteria, but that within each group, rates are relatively constant. Unfortunately, they do not perform any statistical test, so it is unclear whether this apparent variation is significant, and if it is, whether the apparent differences in rate could be removed by the application of a different method to correct for multiple hits. It is a shame that they do not apply their method to synonymous and non-synonymous sites within protein coding genes; with many more genes they would have more power to determine if there are genuine differences between species.

#### Authors' response

The nature of the data set limits our analyses. In the case of 16 rRNA gene, only the *Buchnera *lineage has multiple calibration points, whereas each of the other lineages is represented by a single point. Therefore, we can describe the range of values and their dispersion but not test statistically the differences between different endosymbiont lineages. Unfortunately, comparisons of synonymous and non-synonymous substitution rates between different symbiont lineages are similarly limited.

#### Reviewer comments

They go on to show that Ka and Ks are correlated across species, in both endosymbionts and free-living bacteria, and that the relationship appears to be different for obligate symbionts and free-living bacteria. Again it is unclear whether this difference is statistically significant, but I suspect it is. Surprisingly the correlation between K16S and Ks is very weak and probably would be non-significant if the regression was not forced through the origin (this seems justified). The fact that the relationship between Ka or K16S, and Ks is different between obligate symbionts and their free-living relatives does suggest that there is unlikely to be a universal clock involving either Ka or K16S. They also show that the relationship between Ka and K16S is very similar for obligate and free living bacteria. This suggests that if effective population size is affecting Ka and K16S, then it does so to a similar extent, which would imply that the distribution of fitness effects is similar for proteins and 16S rRNA.

#### Authors' response

We performed the statistical tests suggested by the reviewer and have revised the Results and Methods sections accordingly.

#### Reviewer comments

The authors suggest that there might be a molecular clock within a group of bacteria for certain genes and that to determine the suitable genes one should run relative rate tests to exclude inappropriate data. However, one has to be careful in doing this, because as Bromham et al. showed, this can still leave you with biased estimates, because methods to detect rate heterogeneity are weak (J. Mol. Evol. 50, 296).

#### Authors' response

We have revised the paragraph to address this issue.

### Reviewer's report 2

Dr Simonetta Gribaldo, Institut Pasteur, Paris Cedex 15, France

#### Reviewer comments

This is an interesting paper dealing with an important issue, i.e. the possibility of dating the age of bacteria and different bacterial lineages. The answer is that there is no universal clock for bacteria: not for 16S nor for conserved orthologues. The heterogeneity of evolutionary rates across lineages is indeed a well-known phenomenon and one of the most important issues in phylogenetic reconstruction.

For molecular dating of eukaryotes, many problems have been put forward, of which heterogeneity of rates appear to be only one (as reviewed for example in Roger AJ and Hug LA 2006). I am not an expert of the field, but I think that the use of relaxed clocks and accurate models of sequence evolution can overcome the problem of rate heterogeneity?

For molecular dating of prokaryotes, I have the feeling that the major problem lies in the use of global clocks, the absence of clear calibration points and the use of calibration points very far in time, such as the plant/animal split to date the divergence of a specific bacterial lineage, which has produced obvious overestimations in the literature (see papers by Hedges and colleagues). I think that the introduction would benefit of a more complete discussion of past analyses and their potential problems.

#### Authors' response

Although the use of relaxed clocks and more realistic models of sequence evolution can mitigate the problem of rate heterogeneity, the lack of robust calibration points remains a major challenge to established a timescale for bacterial evolution. We agree with the reviewer's comments and have expanded the introduction as suggested.

#### Reviewer comments

On a more specific issue, is Ka/Ks applicable to large evolutionary distances such as those analyzed here considering molecular saturation?

#### Authors' response

We selected pairs of genomes that are sufficiently, but not excessively, diverged so that we would obtain reliable estimates of *K*_a _and *K*_s_. Of the 42 selected genome-pairs, the median genome-wide *K*_s _values ranged from 0.11 to 1.29 (average = 0.6), and *K*_a _values ranged from 0.006 to 0.058 (average = 0.03). This range of *K*_s _values suggests that saturation is unlikely to cause strong bias in our analysis.

#### Reviewer comments

The authors suggest that it may be possible to date specific lineages once at time by using genes that evolve uniformly among the group (local clock). This suggestion is correct, but I would have expected that the authors move over to show a practical example. However, even in the presence of such a clock, how would the authors deal with the absence of clear calibration points? Finally, this is a short interesting contribution on the problem of molecular dating for prokaryotes, which nevertheless leaves me hungry for more.

#### Authors' response

The *Buchnera*-aphid system shows the feasibility of using local clocks to date bacterial evolution. Unfortunately, there is a paucity of accurate calibration points for free-living bacteria. We have expanded the last paragraph in the Discussions to address this issue.

#### Reviewer comments

Each paragraph is very short and ends a bit brutally, I would suggest merging results and discussion, and then write a conclusion session.

#### Authors' response

Perhaps we are being overly traditional, but our manuscripts typically partition the Results and Discussion, and do not include a Conclusions section. The comprehensive abstracts required of *Biology Direct *articles seem to reduce the need for a separate Conclusions section.

#### Reviewer comments

Page 3: when referring to past analyses on dating of bacteria I think it would be the right place to cite Hedges (1,2).

#### Authors' response

We have added the suggested citations.

#### Reviewer comments

Page 4: a large audience would benefit for a clear definition of calibration point.

#### Authors' response

We have revised the sentence to clarify the definition.

#### Reviewer comments

Page 5: could you please mention in the text the 6 taxa, what phyla they belong to, and a rapid overview of their associations?

#### Authors' response

We have updated the legend of Figure 1 to provide an overview of symbiont-insect associations.

#### Reviewer comments

Page 6: it may be better to say that the 42 pairs of bacterial genomes were selected based on their evolutionary distance rather than on their sequence divergence, since it may give the impression of circular reasoning.

#### Authors' response

We have revised the sentence accordingly.

#### Reviewer comments

Discussion page 9: that Bacteria were the first cellular organisms is a big statement, in my opinion the issue remains open. By the way, the Archaea are never mentioned in the text, although molecular dating has been done for this domain, could the authors comment?

#### Authors' response

Since our manuscript is not concerned with the priority of any particular Domain, we have revised the sentence accordingly. We did not include Archaea in this paper because the taxon sampling of available genome sequence is still too sparse to perform analyses that involve closely related pairs.

#### Reviewer comments

Additional file 1: it is difficult to understand which are the genome pairs.

#### Authors' response

We have listed the species (or strain) names, along with the NCBI genome project ID, for each of the genomes examined in the Additional file 1.

### Reviewer's report 3

Dr Tal Pupko, Tel Aviv University, Tel Aviv 69978, Israel (nominated by Dr Dan Graur, University of Houston, Houston, Texas, United States).

This reviewer provided no comments for publication. The authors are grateful to the reviewer and made several changes to the manuscript based on points raised by this reviewer.

## Supplementary Material

Additional file 1 **Table S1**. List of genome-pairs analyzed. Click here for file

Additional file 2 **Table S2**. List of single-copy genes that are conserved in all genomes analyzed. Click here for file
